# Best quality vs. sex selection – an analysis of embryo selection preferences for patients undergoing preimplantation genetic testing for aneuploidy over a 10-year period

**DOI:** 10.1007/s10815-024-03162-1

**Published:** 2024-06-24

**Authors:** Pavan Gill, Christine Whitehead, Marie Werner, Emre Seli

**Affiliations:** 1 IVIRMA Global Research Alliance, IVIRMA New Jersey, 140 Allen Road, Basking Ridge, NJ 07920 USA; 2 Yale School of Medicine, New Haven, CT USA

**Keywords:** Best-quality embryo, Sex selection, PGT-A, IVF

## Abstract

**Purpose:**

Investigate patient preferences in embryo selection for transfer regarding quality versus sex in IVF/ICSI cycles with PGT-A and assess associated clinical implications.

**Methods:**

Retrospective cohort study at a university fertility practice from January 2012 to December 2021. Included were patients undergoing single frozen euploid transfers with at least one embryo of each sex available. Primary outcomes were preference for embryo selection (quality vs. sex) and sex preference (male vs. female). Trends over 10 years were evaluated and clinical outcomes, including clinical pregnancy rate (CPR), sustained implantation rate (SIR), and live birth rate (LBR), were compared.

**Results:**

A total of 5,145 embryo transfer cycles were included; 54.5% chose the best-quality embryo, while 45.5% selected based on sex. Among those choosing based on sex, 56.5% chose male embryos and 43.5% chose female. Preference for quality remained consistent over the decade (*p* = 0.30), while male embryos were consistently favored (*p* = 0.64). Best-quality embryos had higher grades (*p* < 0.001). Clinical outcomes were similar between groups (CPR: 74.4% vs. 71.9%, *p* = 0.05; SIR: 64.9% vs. 63.4%, *p* = 0.26; LBR: 58.8% vs. 56.7%, *p* = 0.13), and between male and female embryo selections.

**Conclusions:**

Sex selection remains common, with 45.5% selecting embryos based on sex, predominantly favoring males. This trend persisted over 10 years, with comparable clinical outcomes regardless of selection criteria.

## Introduction

The use of preimplantation genetic testing for aneuploidy (PGT-A) in patients undergoing in vitro fertilization (IVF) has significantly increased in the United States with nearly half of patients opting in for PGT-A in 2018 [1]. While the intention of PGT-A is to identify the presence or absence of whole chromosome aneuploidy [2, 3] and to promote single euploid embryo transfer [4], many fertility clinics in the United States also report on the chromosomal sex of the tested embryos, offering the option to then proceed with transfer of the best-quality embryo regardless of sex or to proceed with transfer of a euploid embryo of a preferred sex. In a recent survey of nearly 500 ART clinics across the US, 73% of clinics offered sex selection, despite lack of medical indication. Family balancing (having a child of the opposite sex of previous children) was reported as the most common indication for sex selection across clinics [5]. Another study evaluating how often patients opted for sex selection in IVF and PGT-A cycles pre- and post-delivery of their first child found that sex selection was a greater priority for a subsequent child [6]. While only 32.4% of patients selected for sex in the transfer that resulted in the first live birth, 62% selected for sex when returning for a second child (opting for transfer of the opposite sex of the first child in 81% of cycles) [6].

The American Society for Reproductive Medicine (ASRM) has provided some guidance on this topic. While initial ethics committee opinion reports from the 1990s discouraged the use of sex selection at time of PGT-A for non-medical indications [7], subsequent publications concluded that clinics could permit the use of sex selection for couples seeking gender variety in their families [8, 9]. The most recent ASRM reports have concluded that, while practitioners providing ART treatments should be under no ethical obligation to provide or refuse non-medically indicated sex selection, clinics are encouraged to develop policies regarding whether embryo sex will be incorporated into decisions regarding embryo transfer [10, 11].

While there have been several qualitative studies published exploring perspectives on sex selection in PGT-A cycles [12–14], to our knowledge, there has been only one retrospective study to date that has evaluated patient preferences for sex selection in PGT-A cycles at time of embryo transfer [6]. However, this study only included cycles that resulted in a live birth, had a relatively small sample size, and did not explore trends in preferences over time [6]. The objective of the current study was to investigate if patients who undergo IVF with ICSI and PGT-A have preferences for selecting an embryo for transfer based on best quality vs. sex, to study changes in preferences over a period of ten years, and to determine whether these preferences have clinical implications.

## Materials and methods

### Study design

This was a retrospective cohort study at a single university-affiliated fertility practice. Patients undergoing IVF with ICSI and PGT-A followed by their first single frozen euploid embryo transfer from the beginning of January 2012 to the end of December 2021 (10 years) were included in the study. Only cycles in which at least one embryo of each sex was available at time of transfer were eligible for inclusion. Patients undergoing PGT for monogenic disorders or structural rearrangements were excluded. Transfers of untested embryos and multiple embryo transfers were excluded.

### Ethics approval

Institutional review board approval was obtained to evaluate the retrospective data (Advarra IRB, Pro00027158).

### Participants

Patients underwent controlled ovarian hyperstimulation, most often using the gonadotropin-releasing hormone antagonist protocol. Decisions regarding the type of protocol, dosing of medications and administration of trigger were at the discretion of the patient’s physician, following standard clinic practice. Oocyte retrieval via ultrasound-guided aspiration was performed 36 h after triggering final oocyte maturation. ICSI was performed on all mature metaphase II oocytes. Laser-assisted hatching of the zona pellucida was performed on day three of embryo development. Embryos were then cultured through the expanded blastocyst stage and PGT-A was performed via trophectoderm biopsy prior to cryopreservation. Quantitative PCR was used for PGT-A from 2012 to 2016, next generation sequencing (NGS) was used from 2016 to 2020 and NGS in combination with single nucleotide polymorphism data was used for PGT-A from 2020 to 2021. Embryos were biopsied on day 5, 6, or 7 if they reached the expanded blastocyst stage and were graded at least 4CC or higher based on the modified Gardner scoring system, as per clinic protocol [2, 15]. The blastocysts were evaluated based on rate of expansion (1–6), development of the inner cell mass (A, B, C) and the trophectoderm (A, B, C).

The ICM and TE scores were the primary features used to decide the best embryo for transfer. The expansion score was also considered but given less weight given all embryos were artificially hatched at our center.

Patients were given results on the ploidy status of their embryos as well as morphologic grading. They were also provided information regarding the sex of the embryos unless they chose not to be informed. Prior to proceeding with a frozen embryo transfer, patients were counselled on success rates for each embryo and had the option to proceed with transfer of the best-quality embryo regardless of sex or they could elect to transfer a male or female embryo if they had a preference. If they preferred to proceed with transfer of male or female embryo, the best-quality embryo of the preferred sex was selected for transfer. Single, euploid embryo transfer was performed in a subsequent cycle after obtaining adequate endometrial proliferation via a programmed or modified natural cycle protocol. Progesterone supplementation was continued until ten weeks gestational age.

### Outcome measures

The primary outcome of the study was patient preference for prioritization based on best quality or sex selection at time of embryo transfer, evaluated over the ten-year study period. For those patients who opted for a sex preference, trends in the desire for a male or female embryo were also evaluated during the study period. Secondary outcomes included clinical pregnancy rate (CPR), defined as the presence of an intrauterine gestational sac and yolk sac, sustained implantation rate (SIR), defined as the presence of an ongoing pregnancy with a fetal heartbeat beyond eight weeks gestational age and live birth rate (LBR). These clinical outcomes were compared between groups based on prioritization of best qualify vs. sex preference at time of transfer.

### Statistical analysis

Mean and standard deviation were used to describe descriptive data. Pearson’s chi-square test was used for comparison of categorical variables. *T*-test was used for comparison of continuous variables. Chi-square test for trend was performed to evaluate preferences in embryo for transfer over time. Significance was accepted at *p* < 0.05. Statistical analyses were conducted using SPSS statistical software Version 27.0 (Armonk, NY: IBM Corp.).

## Results

There were 5,145 frozen single euploid embryo transfer cycles included for analysis in the study. The mean age at time of oocyte retrieval was 33.9 ± 4.1 and the age at time of embryo transfer was 34.8 ± 4.2 years. The median number (interquartile range) of euploid embryos available for transfer was 5 (3–7). On average, there were 2 (1–4) male embryos and 2 (1–4) female embryos available. The mean endometrial thickness was 9.8 ± 2.2 mm prior to starting progesterone supplementation. Table 1 describes the baseline characteristics of patients based on transfer preference (best quality vs. sex preference). While the female age at time of embryo transfer was statistically higher (35.1 ± 4.4 vs 34.7 ± 4.0 years, *p* < 0.001) in the sex preference group as was the endometrial thickness (9.8 ± 2.2 vs. 9.7 ± 2.2, *p* = 0.04), these differences were not clinically relevant.

**Table 1:** Baseline characteristics based on embryo preference

Characteristic (*mean* ± SD)	Best quality preference (*n* = 2804)	Sex preference *N* = 2341	*P* value
Female age at embryo transfer in years	34.7 ± 4.0	35.1 ± 4.4	< 0.001
Number of euploid embryos available	5.6 ± 3.3	5.8 ± 3.4	0.05
Male	2.8 ± 2.0	2.9 ± 2.0	0.19
Female	2.8 ± 2.0	2.9 ± 2.0	0.04
Female age at time of oocyte retrieval in years	34.0 ± 3.9	33.9 ± 4.2	0.52
Endometrial thickness prior to initiating progesterone in mm	9.7 ± 2.2	9.8 ± 2.2	0.04
Embryo grade, *n (%)*			
Expansion			< 0.001
4	742 (26.5)	713 (30.5)	
5	1239 (44.2)	913 (39)	
6	823 (29.4)	715 (30.5)	
Inner cell mass			< 0.001
A	1456 (51.9)	915 (39.1)	
B	1302 (46.4)	1331 (56.9)	
C	46 (1.6)	95 (4.1)	
Trophectoderm			< 0.001
A	1304 (46.5)	863 (36.9)	
B	1405 (50.1)	1292 (55.2)	
C	95 (3.4)	185 (7.9)	

**Fig. 1:**
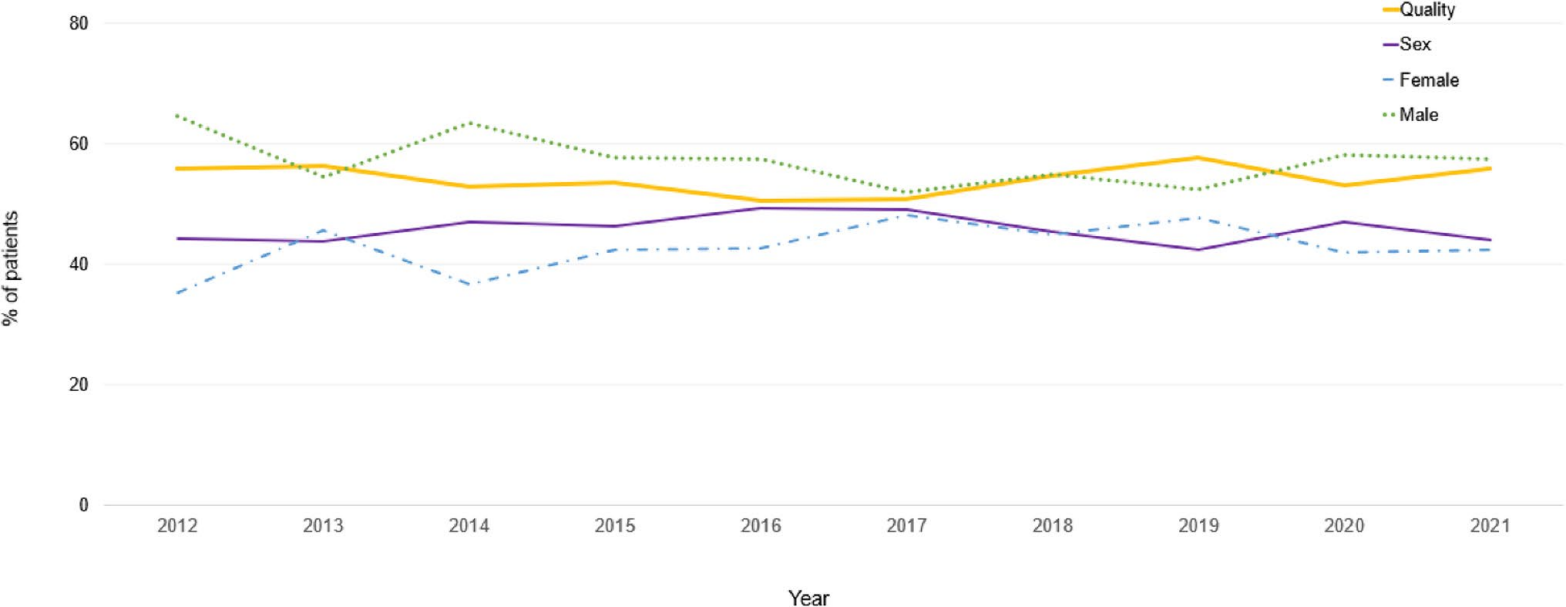
Trends in preferences for embryo transfer over 10 years

More than half of the patients, 54.5% (*n* = 2,804), selected the best-quality embryo for transfer and 45.5% (*n* = 2,341) selected an embryo for transfer based on sex preference. Among those that opted for a specific sex, 56.5% (*n* = 1,324) selected a male embryo for transfer and 43.5% (*n* = 1,017) selected a female embryo. Embryo preferences were also evaluated by year over the 10-year study period. A greater proportion of patients consistently selected the best-quality embryo for transfer over sex preference, with no significant difference in preference over the decade (*p* = 0.302). Similarly, among those who transferred based on sex preference, a male embryo for transfer was consistently preferred over the ten years with no significant difference in preference over the years (*p* = 0.637). Figure 1 describes the trends in embryo transfer preferences over the 10 years.

**Fig. 2:**
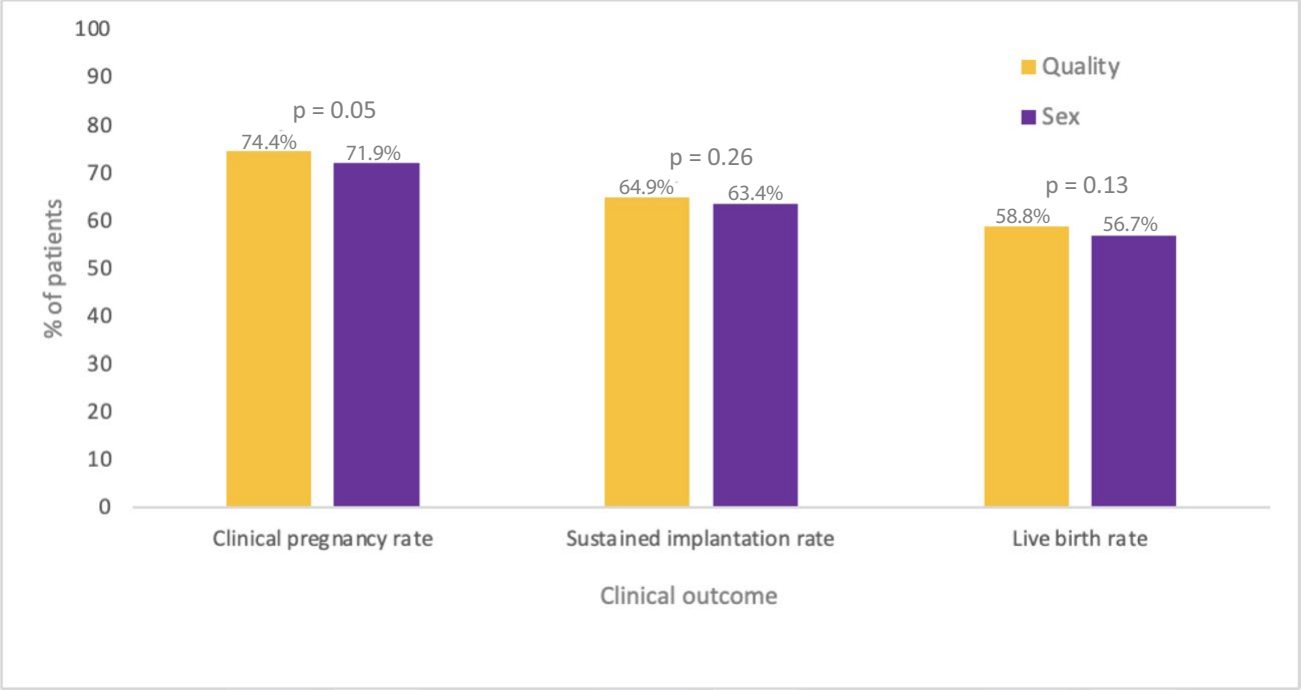
Clinical outcomes based on embryo preference (best quality vs. sex preference)

Clinical outcomes were evaluated based on embryo transferred as shown in Fig. 2. The CPR (74.4% vs. 71.9%, *p* = 0.05), SIR (64.9% vs. 63.4%, *p* = 0.26), and LBR (58.8% vs. 56.7%, *p* = 0.13) were similar between the groups. Outcomes were also compared among those who had a male vs. female embryo preference. There was no significant difference in the expansion score (*p* = 0.81), ICM (*p* = 0.09), there was a higher TE score among the male embryos (*p* =  < 0.001). However, clinical outcomes remained similar in both groups. The CPR was not statistically different between the two groups (72.1% vs 71.7%, respectively, *p* = 0.82). Similarly, the SIR (64.4% vs. 62.1%, *p* = 0.28) or the LBR (57.9% vs. 55.3%, *p* = 0.22) were also not different between the two groups.

## Discussion

In our study of patients who underwent IVF with ICSI and PGT-A testing who had at least one euploid embryo of each sex available at time of transfer, most preferred to proceed with transfer of the best-quality embryo, regardless of sex. However, 45.5% opted for a sex preference at time of transfer. These findings persisted across a 10-year period.

Our analysis found that male embryos were preferred over female embryos among patients who opted for sex selection at time of transfer. These findings are consistent with another large retrospective cohort study completed using SART data which found cycles using preimplantation genetic testing had a substantially higher male to female live birth ratio [16]. In the same study, they reported that the number of cycles using PGT-A for elective sex selection increased by 66% from 2014 to 2016 [16]. However, in our study which looked at global preferences across 10 years, we did not see a trend towards an increase or decrease in preference for sex selection over time. We found that transfer of the best-quality embryo was consistently preferred more often than sex selection.

Our study was completed in New Jersey which is a one of the few states in the US with mandated IVF coverage [17]. Prior studies have suggested differences in preferences for elective sex selection in mandated vs. non-mandated states with lower utilization of IVF for elective sex selection in mandated states [16]. Thus, our findings may not be reflective of states without mandated IVF coverage. Further, ART clinics in the Northeast and West coast of the US as well as clinics located in cities with large populations are more likely to offer sex selection [5] and patients in these settings may be more likely to opt for a sex preference. Additionally, while a previous study found that the LBR was significantly lower in transfers that involved sex selection [6], this was not the case in our study in which both the SIR and LBR did not differ based on embryo transfer preference. Our findings may be helpful in reassuring patients undergoing a single euploid embryo transfer that their decision in opting for best quality or sex should not significantly impact outcomes.

While this study does not explore the ethical aspects of sex selection in PGT-A cycles, there are important considerations that should be explored. These include the balance of further reinforcing societal beliefs regarding gender biases (in particular given the greater preference for male embryos seen in several studies including ours [18]) yet being respectful of the reproductive autonomy of patients who otherwise have very little control in their infertility journey [19].

This study has several strengths, including that it is the largest study to date evaluating patient preferences for embryo transfer in contemporary IVF cycles (e.g., frozen, single embryo transfer utilizing PGT-A). Furthermore, the 10-year analysis period allowed for the opportunity to evaluate trends over time alongside changes in society guidelines/opinion papers. This study however also has limitations. While the objective was to look at global preferences across all patients undergoing their first euploid embryo transfer, it could be helpful to study whether additional characteristics such as race and ethnicity, socioeconomic status [20], and history of prior live births as well as the sex of previous children, could impact patient preferences at time of embryo transfer.

## Conclusion

This study found that sex selection at time of first single, frozen, euploid embryo transfer is important for patients, with nearly half selecting their embryo for transfer based on the sex of the embryo. This trend persisted over a 10-year period and there was a greater preference for a male embryo among those who transferred an embryo based on sex. The CPR, SIR, and LBR were equivalent across both groups, which is reassuring for both patients and providers. Therefore, the decision to choose embryos based on sex does not appear to compromise the overall success rates for patients who may have a preference at time of embryo transfer.
